# Central Serous Chorioretinopathy: A Complication Associated with Behçet’s Disease Treatment

**DOI:** 10.4274/tjo.galenos.2018.83479

**Published:** 2019-02-28

**Authors:** Nur Doğanay, Melike Balıkoğlu Yılmaz, Betül Orduyılmaz, Erdinç Aydın, Ali Osman Saatçi

**Affiliations:** 1 İzmir Katip Çelebi University Faculty of Medicine, Department of Ophthalmology, İzmir, Turkey; 2 Dokuz Eylül University Faculty of Medicine, Department of Ophthalmology, İzmir, Turkey

**Keywords:** Behçet’s disease, central serous chorioretinopathy, steroid

## Abstract

Central serous chorioretinopathy (CSCR) is characterized by a well-defined serous choroidal detachment of the retinal pigment epithelium with one or more focal lesions of the neurosensory retina. Risk factors for CSCR are psychosocial stress, increased endogenous catecholamine, and increased endogenous cortisol. Systemic steroids can cause ocular side effects such as cataract development, increased intraocular pressure, and less frequently the development of CSCR, which can resolve spontaneously with close follow-up and simple treatment modification. CSCR should be considered in patients who complain of worsening vision under steroid treatment for pathologies requiring steroid therapy. In this study we present two patients, one man and one woman, who developed acute CSCR while under systemic steroid treatment for Behçet’s disease.

## Introduction

Behçet’s disease is a multisystemic condition of unknown etiology characterized by chronic, recurrent vasculitis. Ocular and systemic complications can be prevented by controlling the disease with early and effective treatment. Systemic steroids and immunomodulatory agents play an important role in controlling inflammation. Long-term steroid use can lead to systemic side effects such as osteoporosis, predisposition to infection, intestinal ulcers, hyperglycemia, and exacerbated hypertension, as well as severe ocular side effects such as cataract, elevated intraocular pressure and, less frequently, central serous chorioretinopathy (CSCR).^1^

CSCR is characterized by well-defined serous detachment of the neurosensory retina at the macula that may be accompanied by retinal pigment epithelium (RPE) detachment.^2,3^ The pathogenesis is believed to involve choroidal hyperperfusion and RPE barrier dysfunction. Different forms of steroid treatment, such as oral, inhaled, intranasal, intravitreal, and epidural, can cause CSCR.^4^ Here we present two cases, one woman and one man, who developed acute CSCR while under systemic steroid therapy for Behçet’s disease.

## Case Reports

### Case 1

**Figure 1:**
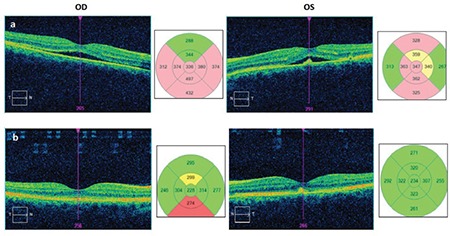
a) Optical coherence tomography shows hyporeflective serous detachment between the subfoveal neurosensorial retina and retinal pigment epithelium in the right and left eyes. In the left eye there is also hyperreflectivity consistent with subfoveal scar associated with serous detachment, b) With steroid tapering, the subfoveal fluid was completely resorbed in both eyes at 6 months, but hyperreflectivity consistent with subfoveal retinal pigment epithelium scar persisted in the left eye

**Figure 2:**
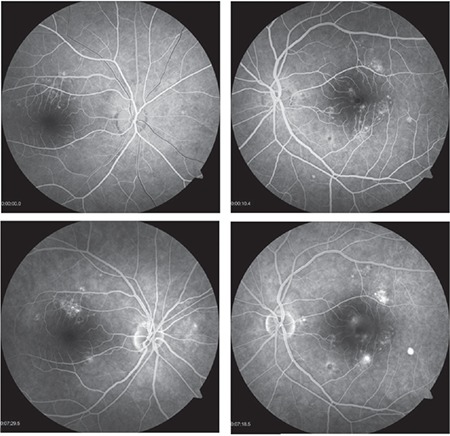
Fundus fluorescein angiography shows multiple focal areas of leakage from the retinal pigment epithelium into the subretinal space in both eyes and macular edema in the left eye

A 44-year-old woman with Behçet’s disease presented to the Uvea and Behçet’s Department of our center complaining of reduced vision in her left eye. Ophthalmologic examination showed her visual acuity was decreased from 1.0 in both eyes to 0.9/0.7 (decimal). Optical coherence tomography (OCT) revealed typical lesions consistent with CSCR in both eyes (Figure 1a). Fundus fluorescein angiography (FFA) showed focal areas of leakage from the RPE into the subretinal space in both eyes (Figure 2). The patient was taking 40 mg/day methylprednisolone, 100 mg/day azathioprine, and 40 mg/day pantoprazole as treatment for Behçet’s disease. She also described panic attack-like symptoms and was referred to the psychiatry department to begin antidepressant therapy. During this time a gradual reduction of her oral steroid dose was planned and antidepressant therapy was initiated. On day 20 of the tapering schedule, her dose of oral methylprednisolone was 32 mg/day and her visual acuity had returned to 1.0 despite persistent bilateral CSCR findings in OCT. On day 90 of the tapering schedule, oral methylprednisolone dose was 16 mg/day, visual acuity remained 1.0 bilaterally, and OCT showed the subfoveal fluid had complete resolved in the right eye but improved only partially in the left eye. Treatment with nepafenac drops 4 times daily was started in the left eye. At 6 months, oral steroid was maintained at 8 mg/day; there were no remaining signs of bilateral serous detachment (Figure 1b) and the patient had full vision in both eyes. No recurrence has been observed during 14 months of follow-up.

### Case 2

**Figure 3:**
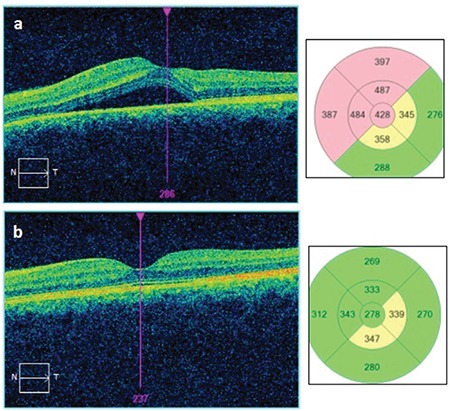
a) Optical coherence tomography revealed a large amount of hyporeflective subfoveal fluid between the neurosensorial retina and retinal pigment epithelium extending superonasally from the subfoveal area, b) OCT taken at month 4 of a steroid tapering schedule showed complete resolution of the serous detachment with no damage to the retinal layers

**Figure 4:**
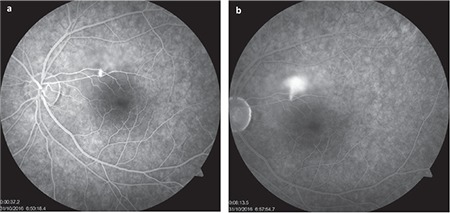
Fundus fluorescein angiography imaging showed a smoke-stack pattern of hyperfluorescence in the superior region of the left macula characteristic of Central serous chorioretinopathy starting in the early phase (a) and increasing in the late phase (b)

A 37-year-old man with Behçet’s disease presented to our clinic with complaints of decreased vision in his left eye. Visual acuity was 1.0/0.6 and anterior segment examination was normal. No pathology was detected on fundus examination in the right eye, while macular OCT showed a typical lesion consistent with CSCR in the left eye (Figure 3a). Increasing hyperfluorescence with smoke-stack pattern was observed in the left macula on FFA (Figure 4). While taking a detailed history, the patient stated he had been prescribed oral methylprednisolone 40 mg/day in the rheumatology department due to arthritis of the left ankle secondary to Behçet’s disease. The patient was referred to the rheumatology department for steroid dose reduction and the psychiatry department due to a stressed psychological state. He was started on oral diazomide 500 mg twice daily and nepafenac drops 4 times daily in the left eye. On day 40 of the steroid tapering schedule, oral methylprednisolone dose was 12 mg/day, visual acuity was improved to 0.7, and OCT showed a significant reduction in subfoveal fluid in the left eye. At 4 months, the methylprednisolone dose was 6 mg/day, his vision was 0.8, and the subfoveal fluid was completely resorbed (Figure 3b). The patient was followed for 16 months. In his final examination, ocular findings were normal with no signs of recurrence.

## Discussion

CSCR commonly affects young/middle-aged men aged 25-55 years and is often unilateral and reversible. About 40% of patients have bilateral disease, though this rate is higher among patients with chronic CSCR.^5^ Similarly, our patients were 44 and 37 years old and represented both sexes. Only the first patient had bilateral CSCR. The primary symptom of CSCR is sudden blurred vision due to fluid leakage and serous detachment in the macula. Metamorphopsia, micropsia, central scotoma, or impaired color vision may also occur.

Risk factors include psychosocial stress, elevated endogenous catecholamine, hypertension, pregnancy, organ transplantation, and obstructive sleep apnea.^6^ Because psychosocial stress was identified as a risk factor in our patients, psychiatric consultation was requested for both of them. Corticosteroids are another important risk factor in the development of CSCR.^7,8^ Steroid use is believed to increase the permeability of choroid capillaries and RPE by inhibiting collagen synthesis and disrupting ion pump function.^9,10^ Glucocorticoids are also known to alter blood-aqueous barrier permeability and disrupt the external blood-retinal barrier by increasing cAMP levels in RPE cells.^11^ Steroids administered by various routes (oral, inhaled, intranasal, intravitreal, epidural) contribute to the development CSCR.^4,12^ In addition, the literature includes reports of a patient under systemic steroid therapy for “retrobulbar neuritis” developing multiple areas of serous retinal detachment in both eyes and a patient misdiagnosed with Vogt-Koyanagi-Harada disease and treated with corticosteroids who actually had atypical bullous CSCR.^13,14^ Both of our patients were receiving oral steroid therapy for Behçet’s disease when they presented with CSCR.

Visual prognosis is generally very good in CSCR and follow-up alone is recommended if there is no underlying pathology. Most patients show spontaneous resolution within 3-4 months.^15^ Patients with steroid-associated CSCR should be followed with their steroid dosage reduced as low as permitted by their disease. In these patients, as in our 2 cases, clinical findings return to normal within a few months, resulting in good visual improvement. The rate of spontaneous resolution is higher in steroid-related cases than with other CSCR etiologies.^16^ Photodynamic therapy (PDT) is a treatment approach commonly used for patients who do not show spontaneous resolution within 3 months, have bilateral or recurrent disease, or who require rapid visual rehabilitation.^17^ However, the effectiveness of PDT may vary for steroid-associated CSCR.^8^ Furthermore, a meta-analysis evaluating the efficacy of anti-vascular endothelial growth factor (anti-VEGF) therapy in CSCR showed that anti-VEGF therapy was not superior to observation at 6-month follow-up in acute cases in which persistent subretinal fluid did not last longer than 3 months.^18^ In an evaluation of comparative studies of chronic cases, however, anti-VEGF therapy was not superior to observation in terms of best corrected visual acuity (BCVA), but there were significant differences between the two groups in terms of central macular thickness (CMT).^18^ In the same meta-analysis, it was reported that non-comparative studies demonstrated significant differences in BCVA and CMT after anti-VEGF therapy at 1, 6, and 12 months follow-up.^18^ Although results have been controversial, intravitreal anti-VEGF injection may be beneficial in chronic CSCR by reducing choroidal vascular hyperpermeability and obstruction.^19^ Both of our patients showed spontaneous resolution.

In conclusion, not only uveitis but also non-uveitic pathologies such as CSCR should be considered when patients present with reduced vision while taking steroids for pathologies such as Behçet’s disease that require steroid therapy. Otherwise, CSCR cases exhibiting atypical clinical features may be difficult to diagnose. CSCR can resolve spontaneously with close follow-up and simple treatment modifications.
